# Post COVID Antimicrobial Resistance Threat in Lower- and Middle-Income Countries: Bangladesh

**DOI:** 10.3389/fpubh.2021.770593

**Published:** 2021-12-17

**Authors:** Ashrafur Rahaman Mahadi

**Affiliations:** Central Medical College, Cumilla, Bangladesh

**Keywords:** COVID-19, antimicrobial resistance (AMR), lower middle income countries (LMICs), Bangladesh, Post COVID

COVID-19, a global pandemic, has put enormous strain on the world's health care systems. One of the most serious issues that clinicians and researchers confront is a lack of effective and easily available COVID-19 treatment possibilities. COVID-19 brought to light the perilous state of our healthcare systems, which had previously flown under the radar from other sectors. This is especially true in low- and middle-income countries (LMICs) and resource-constrained settings, which are less prepared to deal with pandemics or other disasters. Due to lack of knowledge and treatment options for dealing with pandemic in those countries, several antimicrobial medicines are now being used by healthcare workers to treat SARS-CoV-2. Antibiotic overuse and misuse in the treatment of COVID may contribute to the establishment of antimicrobial resistance (AMR). It has the potential to be the next global health disaster, and it has already had an impact on the action to Covid-19 (1–3).

Access to effective antimicrobials is largely unavailable in LMICs, while rates of AMR are expected to expand 4–7 times faster (4). Additionally, because of high rates of improper antibiotic prescribing for COVID-19 patients, treatment interruptions for persons with chronic illness, and broad use of antimicrobial drugs by local populations, COVID-19 has most possibly increased the rate of AMR-related consequences (5).

The lack of awareness about disease outbreaks, misconceptions regarding coronavirus, and social and cultural stigma surrounding the virus have generated a murky dread among the general public throughout the country. As a result, the majority of individuals are frightened of testing positive for coronavirus. When people have COVID-like symptoms, they frequently take self-prescribe antimicrobials such as azithromycin, doxycycline, moxifloxacin, ivermectin, and even specialized experimental medicines from local pharmacies and quacks without seeing physicians and disregarding their possibly harmful side effects. During this SARS-CoV-2 virus outbreak, disinformation on social media about COVID-19, and the availability of treatment prescriptions on numerous Facebook groups encourage individuals to use antibiotics without fully comprehending the dangers. These factors are frequently linked to the misuse of antibiotics during the pandemic (6, 7).

If we analyze the antibiotic prescription rate in Bangladesh, we find that illogical antibiotic prescribing and consumption are highly prevalent among COVID-19 positive individuals in Bangladesh. One study evaluated the antibiotic prescribing rate among SARS-CoV-2 positive patients at a Bangladeshi tertiary COVID-19-dedicated hospital. The findings indicate that 100% of hospitalized patients were getting at least one antibiotic. Antibiotics were prescribed more frequently to individuals with severe illness in general. Ceftriaxone (53.8%), meropenem (40.9%), moxifloxacin (29.5%), and doxycycline (25.4%) were the four most frequently given antibiotics among hospitalized SARS-CoV-2 positive patients (8).

Another study conducted in Bangladesh found that antimicrobial drugs use patterns are significantly high. Cephalosporin, third-generation cephalosporin, macrolide, and azithromycin were the most frequently reported antibiotic classes (9).

A large number of people in our country, regardless of socio-economic status and education, do not consult a registered physician prior to initiating an antibiotic course, frequently relying on retail drugstore's advice. Bangladesh is considered to have a high rate of self-medication since most drugs can be accessed without a prescription from local pharmacies. People can obtain antimicrobials drugs without a prescription from those pharmacies, even in the most distant areas of the region (10).

During the COVID-19 epidemic, this self-medication behavior increased up to 88%. Self-medication for symptoms such as SARS-CoV-2 without doing a COVID-19 test was found to be widespread in Bangladesh during the pandemic period where ivermectin (77%) and azithromycin (54%) were the most commonly self-medicated medicines during the COVID-19 pandemic time (11).

Due to overuse of antibiotics during the pandemic, some drugs might have lost their efficacy against certain specific microbes. Since no new antibiotics are presently being developed for prospective use, the extant antibiotics are losing efficacy, which might have disastrous consequences in the near future. Increasing rates of antibiotic resistance will leave doctors with fewer medication alternatives to manage microbial infectious diseases in the coming years. Misuse of antibiotics will exacerbate the resistance rate and it will be a significant challenge for humanity to overcome (12).

The data of COVID-19 should be used to support future research policies aimed at preventing a future AMR pandemic, which is mainly characterized as a slow-emerging catastrophe which may become more destructive than COVID-19.

In conclusion, lower-middle income countries are at significant risk of future antimicrobial resistance disasters. During this pandemic era, we are nearly disregarding this issue in those countries that are on the verge of a calamity. We will not be able to really address the AMR problem in the world until we develop strong legislation and raise awareness in those countries. To address the rising threat of antimicrobial resistance during this pandemic phase, urgent suitable interventions and preventative measures should be developed from the ground up after identifying the real occurrence and rate of antimicrobial misuse in lower- and middle-income countries including Bangladesh during the COVID-19 pandemic. Additionally, it is essential to implement a surveillance system for antimicrobial medication prescriptions, as well as more strictly enforced sales controls.

## Author Contributions

The author confirms being the sole contributor of this work and has approved it for publication.

## Conflict of Interest

The author declares that the research was conducted in the absence of any commercial or financial relationships that could be construed as a potential conflict of interest.

## Publisher's Note

All claims expressed in this article are solely those of the authors and do not necessarily represent those of their affiliated organizations, or those of the publisher, the editors and the reviewers. Any product that may be evaluated in this article, or claim that may be made by its manufacturer, is not guaranteed or endorsed by the publisher.
